# Top-down factors contribute to differences in insect herbivory between saplings and mature trees in boreal and tropical forests

**DOI:** 10.1007/s00442-020-04659-z

**Published:** 2020-04-20

**Authors:** Elena L. Zvereva, Lucas N. Paolucci, Mikhail V. Kozlov

**Affiliations:** 1 Department of Biology, University of Turku, 20014 Turku, Finland; 2 Setor de Ecologia E Conservação, Departamento de Biologia, Universidade Federal de Lavras, Lavras, CEP: 37200-000 Brazil; 3 Departamento de Biologia Geral, Universidade Federal de Viçosa, Campus Universitário, Viçosa, MG CEP: 36570-900 Brazil

**Keywords:** Arthropod predation, Bird predation, Defensive traits, Ontogenetic changes, Specific leaf area

## Abstract

Ontogenetic changes in herbivory are generally not consistent with ontogenetic changes in defensive traits of woody plants. This inconsistency suggests that other factors may affect ontogenetic trajectories in herbivory. We tested the hypothesis that top-down factors contribute to differences in foliar losses to insects between juvenile and mature trees in tropical and boreal forests. We used artificial caterpillars made of modelling clay to compare predation rates between saplings and mature trees of two common forest species, *Siparuna guianensis* in Brazil (tropical site) and *Betula pubescens* in Finland (boreal site). Leaf area losses to chewing insects in saplings were 2.5-fold higher than in mature trees in both species. Physical plant defences (measured as specific leaf area, SLA) did not differ between saplings and mature trees in the boreal forest, whereas in the tropical forest, SLA was greater in saplings than in mature trees. Attack rates on the model prey by birds were higher in the boreal forest, whereas attack rates by arthropod predators were higher in the tropical forest. Overall, predation rates on model prey were consistently higher on mature trees than on saplings at both sites, but in the boreal site, this pattern was primarily driven by birds, whereas in the tropical site, it was primarily driven by arthropod predators. We conclude that the effect of predation on herbivorous insects may considerably contribute to ontogenetic differences in herbivory, but the relative roles of different predatory groups and of top-down and bottom-up factors may vary between environments.

## Introduction

Plant ontogeny is one of major factors influencing plant–herbivore interactions at both ecological and evolutionary time scales (Boege and Marquis 2005). Much effort has been made to understand ontogenetic trajectories in plant anti-herbivore defensive traits (reviewed by Boege and Marquis 2005; Barton and Koricheva 2010; Massad 2013; Barton and Boege 2017), but despite the study of dozens of plant species, drawing definitive conclusions remains difficult.

Importantly, ontogenetic patterns in plant defensive traits were found to be inconsistent with ontogenetic patterns in herbivory for woody plants (Barton and Koricheva 2010). This inconsistency suggests that factors other than chemical and physical plant defences substantially contribute to the ontogenetic changes in foliar damage imposed by insects on plants. For example, Zverev et al. (2017) found that plant size may explain the level of insect chewing damage on downy birch and, therefore, plant size, affecting plant apparency, may be considered as a defensive trait changing through plant ontogeny. The levels of plant damage may also depend on the strength of the top-down control of insect herbivores (Mäntylä et al. 2011). However, the effects of the third trophic level are only rarely accounted for in explanations of the differences in herbivory between plant ontogenetic stages (but see Boege and Marquis 2006).

A meta-analysis (Mäntylä et al. 2011) showed no differences in bird exclusion effects on plant damage by insect herbivores between mature plants and saplings. However, the lack of significant differences revealed by this meta-analysis may be explained by the considerable variation among the included studies and among the studied plant species, which may exceed the variation between plant ontogenetic stages. The intensity of bird predation on herbivorous insects on trees of different ages has only rarely been compared within one site, and the outcomes of the existing studies are contradictory. In tropical forests, the effect of bird exclusion on insect herbivory was considerably higher in tree canopies than in saplings in some studies (Van Bael et al. 2003; Van Bael and Brawn 2005), while other studies did not find any differences in predation between plant age classes (Boege and Marquis 2006). In temperate forests, the highest predation within a vertical gradient was observed on saplings (Aikens et al. 2013).

Predatory groups other than birds can substantially contribute to the mortality of herbivorous insects and, consequently, to differential patterns of herbivory across plant ontogeny. Arthropod predators are especially abundant in the tropics (Floren et al. 2002; Sam et al. 2015), and they impose higher predation pressure on herbivorous insects than do vertebrate predators, at least in the forest understorey (Roslin et al. 2017). In tropical forests, the abundance of arboreal arthropod predators was higher in the understorey than in tree canopies (Basset et al. 2015). In another study, ants were considerably more abundant on saplings than on mature trees (Basset 2001). Similarly, in a temperate forest, Aikens et al. (2013) found lower invertebrate predation in the canopies of mature sugar maple trees than on conspecific saplings. Nevertheless, studies comparing predation on juvenile and mature trees by arthropod predators are scarce, despite the importance of accounting for the effects of arthropod predators when estimating overall differences in predation.

Vertebrate insectivores can consume both predatory and herbivorous arthropods (Mooney et al. 2010), and bird exclusions can increase the number of arthropod predators (Maguire et al. 2015). Therefore, a negative effect of vertebrate predators on herbivores can be counterbalanced by simultaneous suppression of the arthropod predators of those herbivores (Polis and Holt 1992). As a result, a negative correlation between predation rates by birds and arthropods may considerably influence the resulting estimate of overall top-down control on insect herbivory on saplings and mature plants.

We used modelling clay caterpillars to test the hypothesis that predation pressure from birds and arthropods on herbivorous insects differs between mature trees and conspecific saplings, thereby potentially contributing to ontogenetic changes in herbivore damage. We tested whether differences in leaf losses to insects between saplings and mature trees would follow the pattern in predation rates by birds and arthropods—i.e. a tree age class with higher predation rates would suffer lower herbivore damage. To test this prediction, we measured the damage imposed by chewing insect herbivores and the attack rates on model prey on mature and juvenile trees at two study sites located in tropical and boreal forests. Finally, we controlled for potential effects of ontogenetic changes in plant quality on herbivory by measuring the specific leaf area (SLA), which, along with other mechanical leaf properties, is a better predictor of field herbivory than are concentrations of plant defensive compounds or leaf nutrients (Caldwell et al. 2016; Mediavilla et al. 2018).

## Materials and methods

### Study sites and plant species

We established our experiment at two sites, one in a tropical forest and one in a boreal forest. The tropical site was located in a secondary seasonal semideciduous Atlantic rainforest (Mata do Paraíso Reserve, Viçosa, Minas Gerais, Brazil; 20°48′S, 42°51′W). This is a dense tropical forest dominated by *Bauhinia forficate*, *Piptadenia gonoacantha*, *Anadenanthera macrocarpa* and *Siparuna guianensis* (Marangon et al. 2007), with a shady (5.6% of average canopy openness) understorey. The boreal site was located in a sparse mixed-managed forest near Turku, South-Western Finland (60°32′N, 22°33′E). The forest is dominated by Scots pine (*Pinus sylvestris*), Norway spruce (*Picea abies*) and downy birch (*Betula pubescens*), with abundant birch regrowth. Canopy openness at the boreal site was 58% (Zvereva and Kozlov, unpublished data).

For the experiment, we selected tree species which were common in our sites: downy birch, *Betula pubescens* Ehrh., in Finland and negramina, *Siparuna guianensis* Aublet, in Brazil. Downy birch, a deciduous tree, is widely distributed in Eurasia and is common in most types of boreal forests. This birch is damaged by several hundred different insect species (Atkinson 1992). Negramina, an evergreen tree, is a typical inhabitant of Neotropical forests; however, the data on insects feeding on this plant species are fragmentary and include records of two butterfly species (Robinson et al. 2010), six galling insects (de Araújo et al. 2015) and leaf-cutter ants (Costa et al. 2017).

### Assessment of herbivory

Herbivory was assessed on plant individuals other than those used for measurements of predation rate soon after completion of the predation experiment (in August in Finland and in April in Brazil). Both saplings and mature trees were the same size as those used for measuring predation; they were selected on a “first found, first sampled” basis, but none were closer than 5 m apart. We surveyed 10 plants of each age class in Brazil and 5 mature trees and 18 saplings in Finland. From each mature tree, we collected haphazardly selected low canopy branches, with approximately 100 leaves in total, at the same height where the caterpillar models were attached (i.e. at a height of 1.5−2 m); from saplings, all leaves were collected.

In the laboratory, the leaves on each sapling/branch were counted, and each leaf was examined for the presence and extent of insect damage. Following a widely used methodology (Alliende 1989; Kozlov et al. 2015), each leaf was assigned to one of the damage classes according to the percentage of the area of the leaf lamina consumed by chewing insects: 0% (intact leaves), 0.01–1%, 1–5%, 5–25%, 25–50%, 50–75% and 75–100%. The leaf area lost to insects (AL) was calculated for each plant, as follows: the numbers of leaves in each damage class were multiplied by the respective median values of the damaged leaf area (i.e. 0 for intact leaves, 0.5% for the damage class 0.01–1%, 3% for the damage class 1–5%, etc.); the obtained values were summed for all damage classes and divided by the total number of leaves (including undamaged ones) in a sample. We calculated the percentage of leaf area removed from a damaged leaf (ADL) by dividing the sum of the leaf-specific damage (calculated as described above) by the number of damaged leaves. We also calculated the proportion of damaged leaves (PDL) in each plant as the number of leaves bearing any traces of insect feeding divided by the total number of leaves in a sample. These three plant damage measures are related as follows: AL = ADL × PDL.

### Measurement of specific leaf area

We measured SLA in late June 2018 in ten mature plants and ten saplings of each study species. For the analysis of SLA, we used plant individuals other than those used for exposing artificial caterpillars, because additional damage imposed by sampling leaves for SLA could have affected both herbivory and predation, especially in small seedlings. From each plant, we sampled two current-year leaves that had already completed their growth and we avoided generative shoots and leaves damaged by herbivores. From each leaf, we cut two disks (12 mm diameter in Finland and 16 mm in Brazil) avoiding thick veins. The disks were dried for 24 h at + 80 ºC and then weighed to the nearest 0.1 mg. The SLA was calculated as the leaf disk area divided by its weight.

### Assessment of predation rates

Twenty mature trees (> 3 m tall) and 20 small saplings (30–50 cm tall) were haphazardly selected in each study site; individual plants were at least 5 m apart. Model caterpillars, which were made from non-toxic green modelling clay (Newplast, Newclay Products, UK), were 25–30 mm in length and 4–5 mm in diameter and were built over a wire 0.3–0.5 mm in diameter. These models were attached along thin branches of each of 40 plants per site, 1 model per plant, on 23 May 2018 in Finland and on 16 November 2018 in Brazil. The models were placed in the outer part of the crown at a height of 1.5–2 m in mature trees and at about the middle height of the saplings. The records were conducted at 7–10 day intervals, with the total duration of the experiment 112 days in Finland and 95 days in Brazil. Thus, our observation period covered the entire vegetation season in Finland and the larger part of the rainy season in Brazil; it included periods of high bird breeding activities in both sites, and its length allowed us to account for seasonal variations in bird predation rates (described e.g. by Remmel et al. 2009; Molleman et al. 2016). Each model was classified as attacked or not attacked by each of four groups of predators (arthropods, birds, mammals, reptiles); attribution of damage marks followed Low et al. (2014). Then, any models that had damage marks were remoulded or replaced if the damage was severe.

### Statistical analysis

All our analyses explored the effects of site, plant age and their interaction (fixed effects) on predation, herbivory and SLA. Random effects were the observation period nested within individual tree (in the analysis of predation) and tree nested within site and age group (in the analysis of SLA). We analysed the three measures of predation (by birds, by arthropods, and by all predators combined) using a logistic regression analysis (with a binary error distribution and the logit link function). The three measures of herbivory and SLA were analysed by ANOVA. We used SAS GLIMMIX procedure for all analyses (SAS 2009). To facilitate accurate *F* tests of the fixed effects, we adjusted the standard errors and denominator degrees of freedom by the latest version of the method described by Kenward and Roger (2009).

## Results

**Fig. 1:**
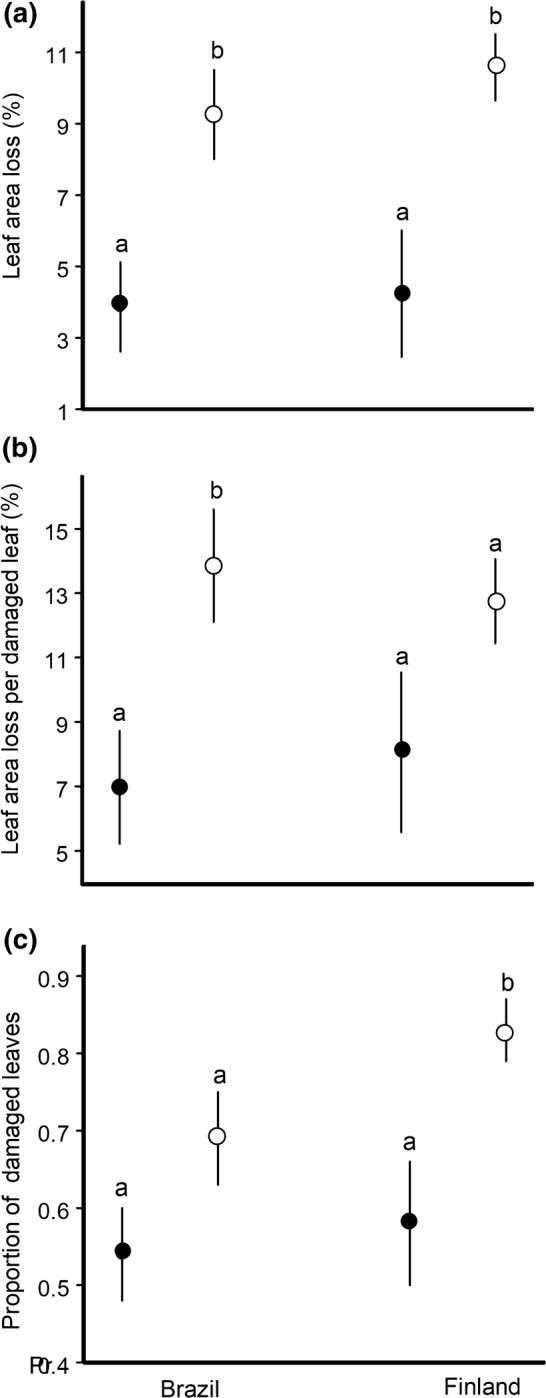
Estimated marginal means (± SE) for (**a**) overall leaf area loss to insect herbivores, (**b**) leaf area loss from a damaged leaf and (**c**) proportion of damaged leaves on mature plants (filled circles) and saplings (empty circles) of *Siparuna guianensis* (Brazilian site) and *Betula pubescens* (Finnish site). The values marked with different letters differ significantly (*P* < 0.05) from each other within a site

The overall losses of plant foliage to chewing insect herbivores were similar in our study sites (6.56% in Brazil and 7.41% in Finland) but were consistently higher in saplings than in mature trees at both study sites. The area consumed from a damaged leaf and the proportion of damaged leaves were also both higher in saplings than in mature trees (Table 1, Fig. 1b, c), but our sites differed in terms of the relative importance of these two components of overall leaf loss. The difference between saplings and mature trees in the area consumed from a damaged leaf was significant in Brazil, but not in Finland (Fig. 1b), while the difference in the proportion of damaged leaves was significant in Finland, but not in Brazil (Fig. 1c).

**Table 1:** Effects of study site and plant age on plant losses to herbivorous insects (ANOVA, type III tests) and on the attack rates on clay caterpillars by predators (logistic regression analysis with a binary error distribution and the logit link function)

Source of variation	Site		Plant age		Site × plant age	
	Test statistics	*P* value	Test statistics	*P* value	Test statistics	*P* value
Overall leaf area loss	*F*_1, 39_ = 0.40	0.53	*F*_1, 39_ = 19.2	< 0.0001	*F*_1, 39_ = 0.13	0.72
Leaf area loss from a damaged leaf	*F*_1,39_ = 0.00	0.99	*F*_1,39_ = 9.55	0.004	*F*_1,39_ = 0.34	0.56
Proportion of damaged leaves	*F*_1,39_ = 2.14	0.15	*F*_1,39_ = 10.37	0.003	*F*_1,39_ = 0.48	0.49
Avian predation rate	*F*_1, 243.1_ = 20.8	< 0.0001	*F*_1, 243.1_ = 5.05	0.026	*F*_1, 243.1_ = 0.52	0.47
Arthropod predation rate	*F*_1, 227.2_ = 43.7	< 0.0001	*F*_1,227.2_ = 6.35	0.012	*F*_1, 227.2_ = 0.79	0.37
Overall predation rate	*F*_1, 219.3_ = 6.67	0.01	*F*_1,219.3_ = 19.9	< 0.0001	*F*_1, 219.3_ = 0.23	0.63
Specific leaf area	*F*_1,36_ = 0.79	0.38	*F*_1,36_ = 3.45	0.07	*F*_1,36_ = 4.25	0.046

**Fig. 2:**
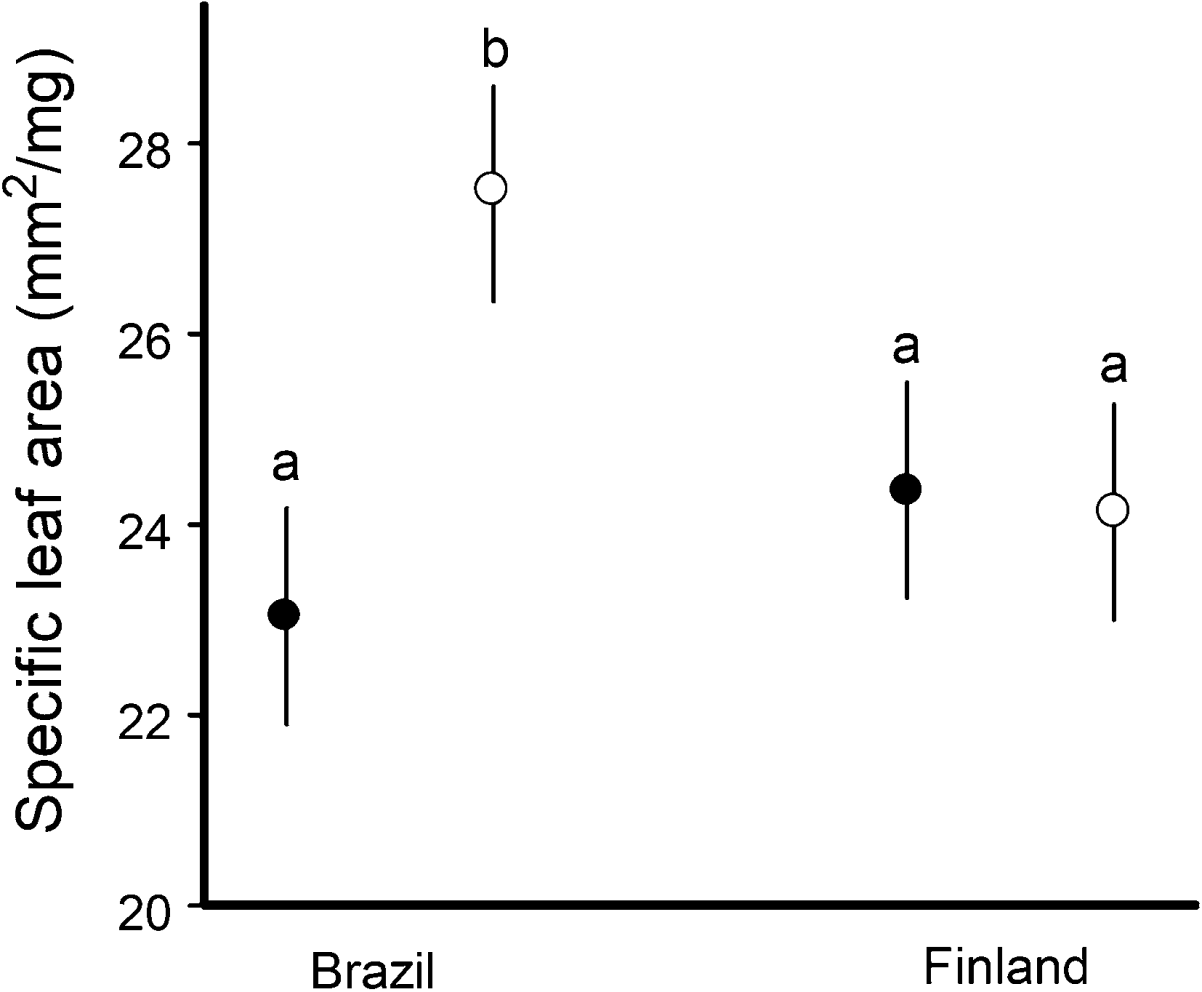
Estimated marginal means (± SE) for specific leaf area of mature plants (filled circles) and saplings (empty circles) of *Siparuna guianensis* (Brazilian site) and *Betula pubescens* (Finnish site). The values marked with different letters differ significantly (*P* < 0.05) from each other within a site

SLA did not differ between *B. pubescens* and *S. guianensis*, but the effect of ontogenetic stage on SLA differed between boreal and tropical sites (Table 1). In the boreal site, saplings and mature plants of *B. pubescens* did not differ in SLA, whereas in the tropical site, the SLA of *S. guianensis* was greater in saplings than in mature trees (Fig. 2).

The vast majority of damage marks on our model prey were classified as having been made by birds or by arthropod predators. Attacks by mammal predators were too rare (on two models in Finland and on three models in Brazil) to analyse them separately, but they were included in the estimate of total predation; no attacks by reptile predators were recorded.

**Fig. 3:**
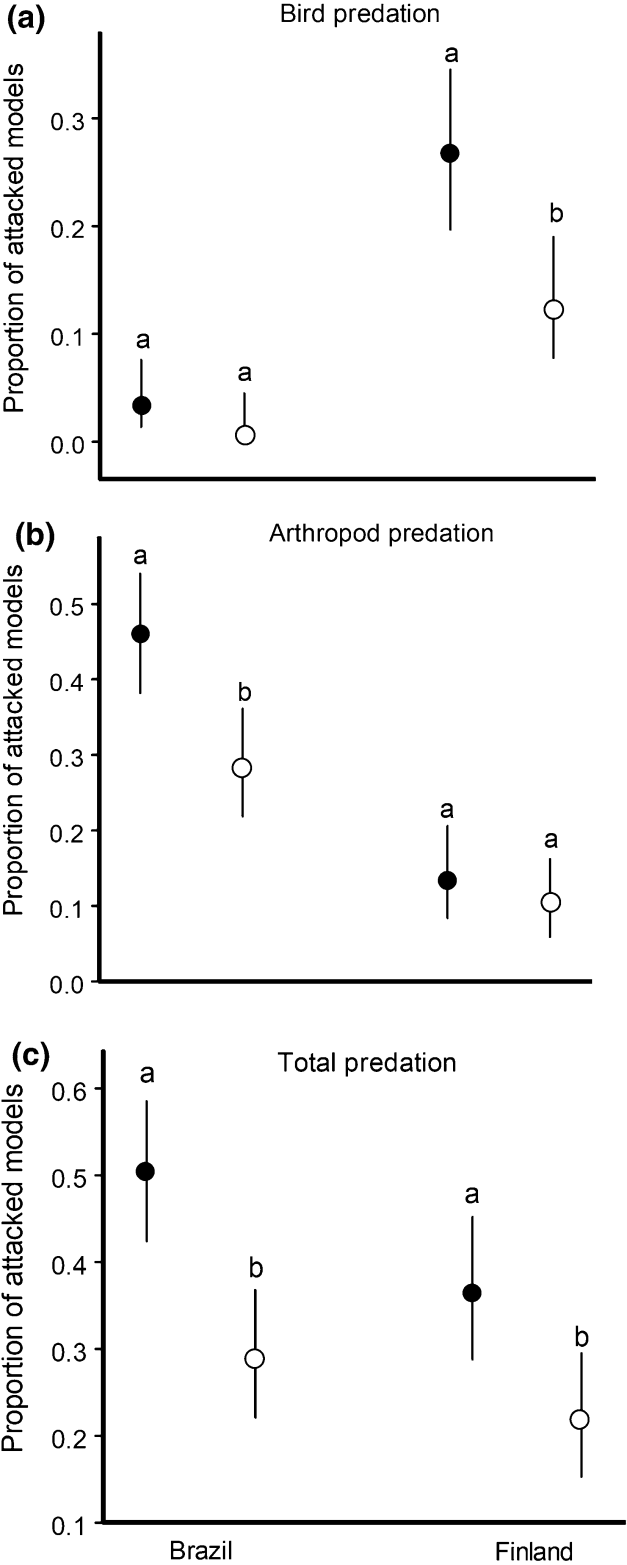
Estimated marginal means (and 95% confidence intervals) for predation rates by birds (**a**), arthropod predators (**b**) and all predators combined (**c**) on modelling clay caterpillars attached to mature plants (filled circles) and saplings (empty circles) of *Siparuna guianensis* (Brazilian site) and *Betula pubescens* (Finnish site). The values marked with different letters differ significantly (Tukey–Kramer test, adjusted *P* < 0.05) from each other within a site

Predation by both birds and arthropods on model prey differed between the study sites (Table 1): attack rates by birds were higher at the Finnish site, whereas attack rates by arthropods were higher at the Brazilian site (Fig. 3a, b). Overall predation was greater at the Brazilian site than at the Finnish site due to a generally higher arthropod predation (Table 1, Fig. 3c).

The proportions of the models attacked by both avian and arthropod predators were generally higher on mature trees than on saplings across both study sites (Table 1), but within-site comparisons revealed that this difference was significant at the Finnish site only for bird predation (Fig. 3a) and at the Brazilian site for arthropod predation (Fig. 3b). The overall predation rate was significantly higher on mature trees than on saplings at both sites (Fig. 3c).

## Discussion

### Bottom-up effects on herbivory

We found higher leaf area losses to herbivorous insects in saplings than in mature trees in natural environments, and this pattern was consistent between tropical and boreal forests. These differences between tree age classes are in line with several other studies (reviewed by Barton and Koricheva 2010); they are usually explained by the increase in resource allocation to defence, in particular to an increase in leaf toughness with plant growth (Stiegel et al. 2017; Castagneyrol et al. 2019). We assessed ontogenetic changes in leaf physical properties by measuring SLA, which not only directly affects leaf palatability for chewing insects, but also positively correlates with other leaf traits (e.g. foliar nitrogen) that enhance plant quality for herbivores (Reich et al. 1999; Write et al. 2004). Hence, we suggest that higher SLA could have contributed to greater herbivore damage on sapling than on mature plants in the Brazilian site. In contrast, but in line with an earlier study (Zverev et al. 2017), birch leaf SLA in our boreal site did not change with plant age. Therefore, physical defences are not likely to explain the ontogenetic changes observed in birch herbivory at the Finnish site.

Our findings suggest that the differences in foliar losses to insects between mature and juvenile plants (Fig. 1a) discovered in our tropical and boreal sites were driven by different mechanisms. In Brazil, higher overall losses in saplings of *S*. *guianensis* were mostly due to a greater amount of area lost from a damaged leaf, whereas in Finland, it was mostly due to a greater proportion of damaged leaves (Fig. 1b, c). Insects feeding on leaves with high physical defence (as reflected by low SLA) exhibit lower consumption rates (Clissold et al. 2009), change their feeding sites twice more often, and spend three times more time moving between feeding sites when compared with insects feeding on less defended leaves (Zvereva et al. 1998). These changes in feeding behaviour may explain the extent of the consumed area in a single leaf: on saplings with a high SLA, chewing insects feed longer on one place than they do on mature plants with a lower SLA, thereby resulting in higher area losses from a damaged leaf. Thus, changes in leaf physical properties are likely to contribute to ontogenetic changes in herbivory in *S. guianensis*, but not in *B. pubescens*.

Changes in leaf physical traits with plant growth may occur due to ontogenetic shifts in defensive strategies (Boege and Marquise 2005; Barton and Koricheva 2010). However, differences in leaf physical properties between juvenile and mature trees may be determined not only by ontogenetic processes, but also by environmental factors, such as light availability. Plants or leaves growing in shade usually have lower toughness/thickness and, conversely, high SLA (Louda and Rodman 1996; Guerra et al. 2010; Kitajima et al. 2016). The understorey of a tropical forest is deeply shaded, so the understorey plants sometimes receive less than 1% sunlight (Kitajima et al. 2016; Messier et al. 2009), in line with low canopy openness at our Brazilian site. Therefore, the differences in SLA between mature plants and saplings in *S. guianensis* may be explained by the different light conditions in understorey versus the tree crown. This hypothesis is supported by the observed decreases in SLA of the understorey *S. guianensis* plants with an increase in forest disturbance (Prado Júnior et al. 2015). In contrast, the saplings of *B. pubescens* in our Finnish site grow under a high light availability, because the forest has high canopy openness due to recent management. The lack of differences in SLA between saplings and trees is therefore in line with their growth in similar light environments.

Chemical defences can also change with plant growth (Boege and Marquise 2005). Our tropical study plant, *S. guianensis*, is rich in sesquiterpenes (Andrade et al. 2015), which may provide anti-herbivore defence (Ferreira et al. 2017; Loureço et al. 2018). We do not have data regarding the ontogenetic changes in the defensive compounds of *S. guianensis*, but two other Brazilian tree species have higher concentrations of sesquiterpenes in saplings than in mature trees (Langenheim et al. 1986; Macedo and Langenheim 1989); this pattern would result in lower herbivory on saplings. Similarly, birch saplings have higher concentrations of some defensive compounds when compared with mature plants (Reichardt et al. 1984). Therefore, ontogenetic changes in chemical defences are unlikely to explain the higher herbivory we observed on saplings in either of our study species.

Bottom-up factors other than host-plant quality for herbivores may also contribute to ontogenetic changes in herbivory. For instance, plant size may be a good predictor of herbivory due to its effect on plant apparency (Castagneyrol et al. 2013; Strauss et al. 2015; Zverev et al. 2017). Accordingly, escaping from herbivory, in particular due to small plant size, is a well-known mechanism of resistance (Boege and Marquis 2005) that provides a first line of plant defence. The effects of apparency on plant damage by insects can explain, in some cases, the lack of correspondence between the levels of plant defences and field herbivory, as has been demonstrated by Barton and Koricheva (2010). However, the plant apparency hypothesis predicts lower herbivory in saplings than in large trees, whereas we observed the opposite pattern; therefore, this explanation is not applicable to our results. The discrepancy between the two studies that compared losses to insects on mature trees and saplings of the same species, *B. pubescens* (Zverev et al. 2017 and this study), indicates that ontogenetic trajectories of herbivory in nature depend on multiple factors and are highly context specific. In particular, Zverev et al. (2017) observed among-site variation in the direction of differences between juvenile and mature plants: although mature birches, on average, suffered more damage than saplings did, three of ten sites showed higher herbivory on saplings than on mature plants, i.e. the same pattern as found in the current study.

Thus, the bottom-up factors alone cannot explain the observed differences in herbivory between saplings and mature trees, although they may have contributed to the ontogenetic differences in herbivory in our tropical site.

### Between-site differences in predation rates

Higher arthropod predation in our tropical site than in our boreal site is in line with other studies, conducted with both natural (Jeanne 1979) and clay model preys (Roslin et al. 2017; Zvereva et al. 2019), which found higher arthropod predation in the tropics than in temperate and boreal zones. Ants were the main arthropod predator in our boreal site; several nests of *Formica aquilonia* were located within the study area, and some of them were as close as two meters from the experimental plants. In the tropics, arthropod predation in tree crowns is also dominated by ants, but their abundance, diversity and activity are considerably higher there than in other environments (Jeanne 1979; Floren et al. 2014; Kaspari and de Beurs 2019). Some researchers raise concerns about whether arthropod predators perceive modelling clay caterpillars as real prey because the models do not possess the chemical cues important for prey recognition by many invertebrate predators (Vet and Dicke 1992). However, the correspondence between the results of studies that measured predation by different methods (cited above) indicate that arthropod predation, or at least predatory activity on herbivorous prey, is indeed higher in tropics than in other biomes. However, the modelling clay prey method is likely to overestimate the differences in arthropod predation between sites that substantially differ in ambient temperatures at the time of model prey exposure due to the shallower indentations, and thus lower visibility, of arthropod attack marks on modelling clay in colder climates (Muchula et al. 2019).

In contrast to arthropod predation, bird predation rates were higher in the boreal site than in the tropical site. This result contrasts with the higher density of insectivorous birds and the greater biomass of arthropods consumed by these birds per hectare in tropical forests relative to temperate and boreal forests (Nyffeler et al. 2018). However, our result is in line with another study, which found lower bird predation on modelling clay caterpillars in three tropical sites than in three boreal sites (Zvereva et al. 2019). These results may be at least partly explained by the high abundance of alternative food in the tropics, such as fruits and non-herbivorous arthropods (e.g. ants and spiders; Floren et al. 2002; Cardoso et al. 2011), which may decrease bird predation pressure upon herbivorous insects.

### Predation and plant ontogeny

The importance of top-down factors in shaping ontogenetic changes in herbivory was suggested long ago (Boege and Marquise 2006; Boege et al. 2011), but this hypothesis received surprisingly little experimental support (but see Van Bael et al. 2003). Our study provides unequivocal experimental evidence regarding the importance of these factors in shaping ontogenetic trajectories in herbivory in natural environments.

Several studies have discovered differences in predatory arthropod abundance and in predation rates on herbivorous insects between tree canopies and understoreys where saplings are growing (Loiselle and Farji-Brener 2002; Van Bael et al. 2003; Ulyshen 2011; Aikens et al. 2013). Some of these studies reported a higher abundance of arthropod predators (Basset 2001) or higher arthropod predation (Loiselle and Farji-Brener 2002) in tree canopies than in understorey vegetation. Both these examples refer to tropical forests, while Aikens et al. (2013) found an opposite pattern in a temperate forest. In tropical forests, where ants are the most abundant arthropods preying on herbivorous insects (Loiselle and Farji-Brener 2002; Sam et al. 2015), the ant community includes many arboreal species (Floren et al. 2002, 2014); therefore, ants dominate in tree canopies. Wasps, the second most abundant group of arthropod predators in the tropics, also prey more in tree canopies than in understoreys (Ulyshen 2011). Thus, our finding of higher arthropod predation on mature trees than on saplings in our tropical site may be explained by a higher abundance and/or activity of ants and wasps in the tree canopies compared with the tropical forest understorey. At the same time, the wood ants dominating our boreal site are mostly epigeic and search for their insect prey both on the ground and in the lower parts of tree canopies, where they tend aphids (Punttila et al. 2004; Domisch et al. 2009). This could then explain the lack of differences in arthropod predation at our boreal site.

Birds attacked our models more frequently on mature trees than on saplings, and the absence of interaction between the site and plant age suggests that this effect occurred at both our sites. We, therefore, suggest that the lack of statistical significance in the differences between the saplings and mature trees in our Brazilian site is explained by the extremely low rates of bird predation. Our result is in line with the bird exclusion study by Van Bael et al. (2003), who found that birds decreased arthropod densities and damage to the leaves of mature plants, but not of conspecific saplings, in tropical forests. Our study, conducted with a different method, indicates that low bird predation on saplings appears to be a general pattern across different forest habitats. This pattern may be related to the higher productivity of canopy branches compared with saplings, because predators are predicted to effectively limit herbivores mainly in areas of high plant productivity (Oksanen et al. 1981). Canopy branches may produce three times as much leaf area per day when compared with understorey saplings, and the overall numbers of potential bird prey are higher on canopy branches than on saplings (Van Bael et al. 2003; Bassett et al. 2015). Accordingly, foliage-gleaning birds foraging in tree canopies are generally more abundant and diverse than are birds foraging in understoreys (Robinson and Holmes 1982; Van Bael et al. 2003; Castaño-Villa et al. 2019).

The difference in bird predation on herbivores between plant ontogenetic stages may be also related to changes in plant size (Boege and Marquis 2005). The optimal foraging theory (Stephens and Krebs 1986) states that predators are expected to minimise the time locating prey while maximising food intake. Hence, even when the density of herbivorous insects is similar on mature and juvenile trees, foraging within the crown of a single mature tree is more advantageous than is visiting numerous saplings with about the same volume of foliage. Additionally, ontogenetic changes in plant architecture may contribute to the observed differences in bird predation: foliage gleaners may have difficulty perching on the thin stems of saplings during foraging.

## Conclusions

We found that herbivorous insects experience a higher predation pressure when located on mature trees than on conspecific saplings, and we concluded that this difference could have contributed to the observed ontogenetic changes in herbivore damage. Our study adds to a very scarce body of evidence for a role of top-down factors in shaping ontogenetic trajectories in herbivory, and the effects of predators may be explained by ontogenetic changes in plant size and architecture. We also found that the relative roles of the two major predatory groups differed between the studied forests: in the boreal forest, a higher predation on mature trees than on saplings was primarily driven by birds, whereas, in the tropical forest, prey mortality was primarily driven by arthropod predators. Ontogenetic shifts in plant defences could also have contributed to variations in herbivory, but in natural environments, differences in light availability between the tree crowns and the understorey may also affect changes in herbivory as plant grows.
